# Molluscum Contagiosum Complicated With an Epidermal Cyst: A Case Report

**DOI:** 10.1002/ccr3.72291

**Published:** 2026-03-26

**Authors:** Ruixian Niu, Wenjin Geng, Yun Zou, Weian Kuang, Jiangtao Zheng, Xunguo Yin, Na Qiao

**Affiliations:** ^1^ Department of Dermatology Qujing Central Hospital of Yunnan Province Qujing China

**Keywords:** epidermal cyst, molluscum contagiosum, viral inclusion body, viral infection

## Abstract

Molluscum contagiosum (MC) is a viral infection that primarily affects pediatric patients, sexually active young adults, and immunocompromised individuals of all ages. Clinically, MC presents as firm, rounded, pink, or skin‐colored papules with a shiny and umbilicated surface. Microscopic histological findings include a crateriform invagination of the hyperplastic epithelium composed of enlarged keratinocytes containing inclusion bodies known as Henderson–Patterson bodies. In some patients, including immunocompetent children, the morphology of MC lesions can vary. Giant MC lesions can mimic cysts, abscesses, or condylomas. Erosive MC lesions can mimic eczema vaccinatum. The papules may also be pearly white or reddish in color. Here, we report a case of a Molluscum contagiosum infection complicated by an epidermal cyst.

## Key Clinical Message

Molluscum contagiosum (MC) often occurs in clusters, and the clinical manifestations of the skin lesions are diverse. Isolated or large lesions are prone to confusion with other skin diseases. Therefore, careful differential diagnosis is necessary. Early detection and accurate diagnosis are crucial to improve patient prognosis.

## 1 Case History and Examination

**FIGURE 1:**
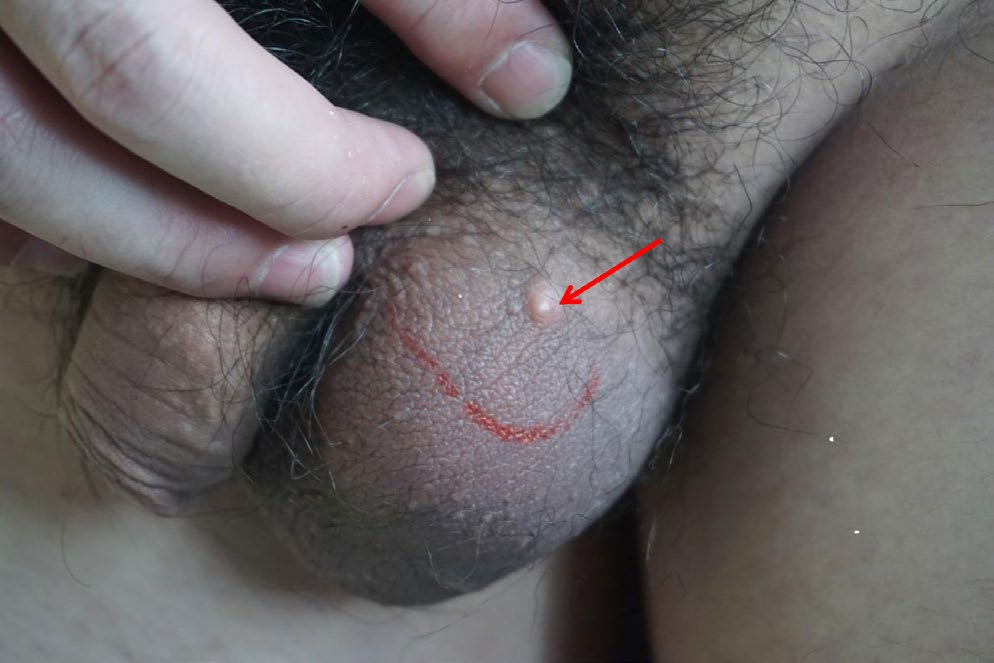
Skin‐colored, shiny papules on the scrotum (indicated by red arrow).

**FIGURE 2:**
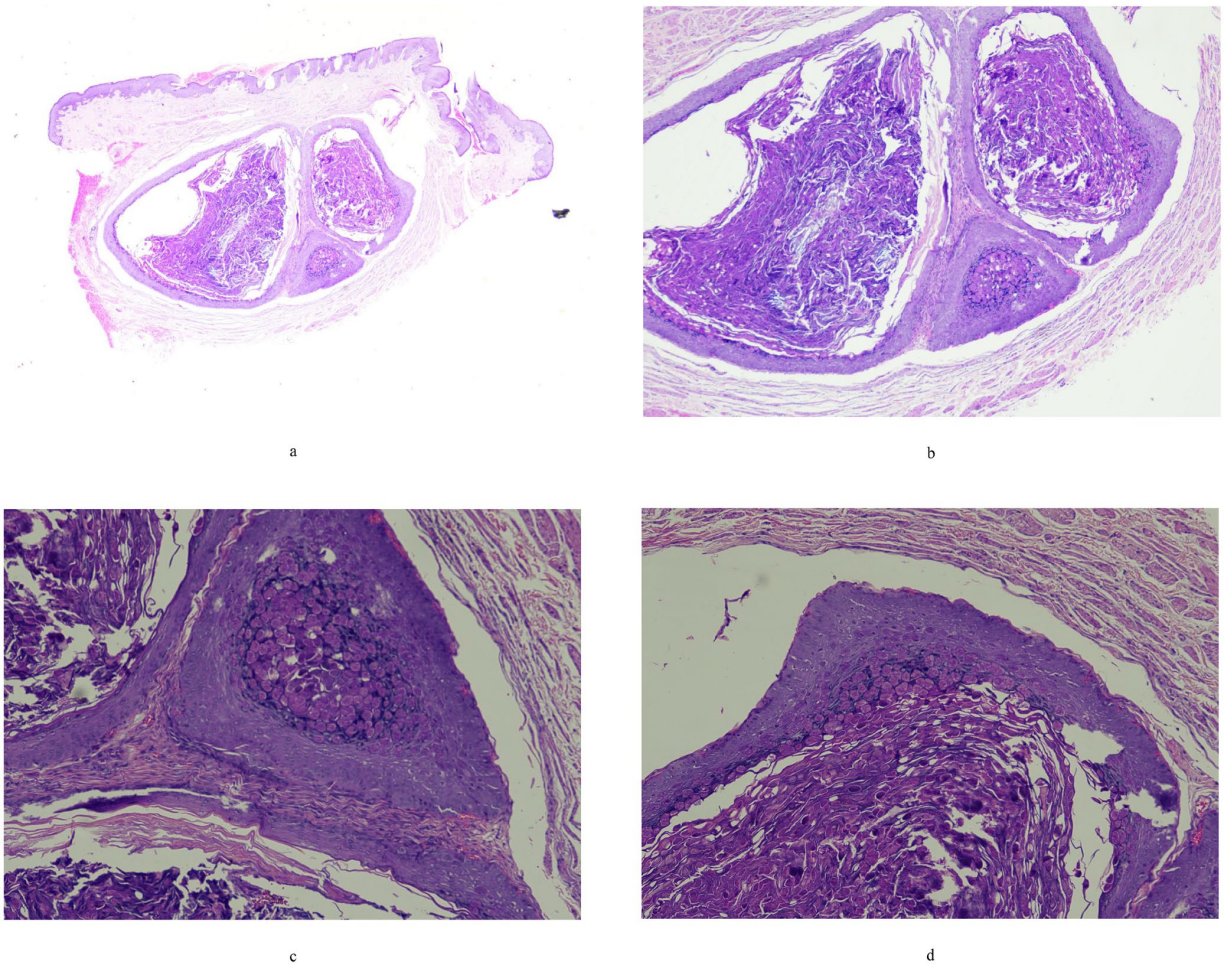
(a) A cystic structure can be observed within the epidermis (HE X 20). (b) Inside the sac, there are visible keratinous substances and viral inclusions (HE X 40). (c) Viral inclusion body (HEX100). (d) Virus inclusion bodies can be observed on the cyst wall (HEX100).

An otherwise healthy 24‐year‐old Chinese Han man presented with a 0.5 × 0.5 cm nodular on the scrotum that had persisted for 1 month. The patient had large skin‐colored nodules on the scrotum without obvious induction and no previous symptoms. He was treated in our outpatient department with topical fusidic acid for a presumed epidermal cyst. After 1 month of treatment with no improvement, the patient revisited our department. Physical examination revealed a 0.5 × 0.5 cm nodular on the scrotum that the lesion was moderately mobile, medium texture, and no obvious tenderness. The remainder of the scrotum exhibited no apparent lesions (Figure 1). Pathological examination of the surgically removed lesion revealed that a cystic structure can be observed within the epidermis, and within the cyst, there are keratinous substances and viral inclusions (Figure 2a–d) [1].

## 2 Diagnosis

Molluscum contagiosum with epidermal cyst.

## 3 Differential Diagnosis

Epidermal cysts, sebaceous gland hyperplasia, pigmented basal cell epithelioma, pigmented cutaneous fibroma and its appendages.

## 4 Treatment

Surgical resection.

## 5 Outcome and Follow‐Up

No recurrence, no similar lesions in other areas.

## 6 Discussion

MC often occurs in immunodeficient patients (such as the patients with malignant tumors and AIDS). Isolated or large skin lesions are easily confused with other skin diseases in the clinic, and comprehensive diagnosis is needed. It should be distinguished from epidermal cysts, pigmented basal cell epitheliomas, pigmented dermatofibromas, and accessory tumors [2].

## 7 Conclusion

The clinical manifestations in this patient were atypical of molluscum contagiosum. This case emphasizes the importance of histopathology for an accurate diagnosis. Therefore, clinicians must be aware that the early detection and accurate diagnosis of this condition are crucial for improving patient prognosis.

## Author Contributions

Ruixian Niu: data curation, formal analysis, and writing the original draft. Wenjin Geng, Yun Zou, Weian Kuang, Na Qiao, and Xunguo Yin: conceptualization, formal analysis, writing – review, and editing.

## Funding

The authors have nothing to report.

## Consent

Written informed consent was obtained from the patient for the publication of this case report and any accompanying images.

## Conflicts of Interest

The authors declare no conflicts of interest.

## Data Availability

Statement will be published alongside your manuscript, if it is accepted for publication.
